# Barriers and Limitations of Employment in Desistance: Exploring the Experiences of Men Leaving Prison in Aotearoa New Zealand

**DOI:** 10.1177/0306624X251352862

**Published:** 2025-07-09

**Authors:** Alice Mills, Grace Low, Cinnamon Lindsay Latimer

**Affiliations:** 1 University of Auckland, Auckland, New Zealand; 2 Oxford Brookes University, UK

**Keywords:** Aotearoa New Zealand, desistance, employment, Māori, Indigenous, colonial context, knifing off

## Abstract

Through narrative interviews with 16 men leaving prison in Aotearoa New Zealand, the current research contributes to the literature on the role of employment in desistance by emphasising some of the barriers to (or limitations of) its desisting effects. In particular, the study emphasises structural constraints in the colonial setting of Aotearoa New Zealand, which can prevent Indigenous Māori men from obtaining stable, rewarding work. The study emphasises how different types of work and work environments can have varying effects on desistance, with some even encouraging reoffending and needing to be ‘knifed off’ to support the men’s desistance journeys. It is argued that those leaving prison should not be encouraged into ‘any’ job, but rather work which provides adequate remuneration, satisfaction, and opportunities to build a positive identity to foster desistance. This may require the state, particularly the Department of Corrections, and employers to facilitate opportunities into suitable employment.

## Introduction

Considerable literature recognises that obtaining employment can promote desistance from crime (Farrall, 2002; Maruna, 2001; Sampson & Laub, 1993). However, such literature typically recognises that it is not ‘any’ job that promotes desistance, but rather stable, meaningful work which provides a liveable wage, and some form of work satisfaction. Those leaving prison face considerable barriers to obtaining work upon release, and available employment tends to be characterised by low pay, seasonality and precarity with limited opportunities for career development and progression (Sheppard & Ricciardelli, 2020). Such precarious work may not have the same (if any) desisting effects as more gratifying work.

The current study seeks to build on the existing literature on employment and desistance in two ways. First, it seeks to explore the barriers to the desisting effects of employment in the colonial setting of Aotearoa New Zealand. Literature that explores desistance in a colonial context generally and the barriers to obtaining work in this context is limited. The current study considers how a sample of 16 men leaving prison in Aotearoa New Zealand (seven of whom identify as Māori, New Zealand’s Indigenous population) experience employment upon release from prison, and the barriers that they face to obtaining stable, quality work. Second, the study seeks to provide further theoretical development concerning the ‘limits’ of employment (or particular types of employment) in supporting desistance from crime. It analyses the ways in which certain types of work can operate as a pathway into offending, and argues that, for some, distancing oneself from certain types of work may be necessary for desistance. The implications of these findings for the provision of employment and related support are discussed.

## Literature Review

### Employment and Desistance

Desistance, or the process of how people with offending histories cease offending, is commonly associated with obtaining employment (Farrall, 2002; Maruna, 2001; Sampson & Laub, 1993). Employment is often recognised as an essential foundation to establish a law-abiding life after prison (Anazodo et al., 2017). The precise mechanisms through which employment influences desistance appear to be many and varied (Godfrey et al., 2007). It may make crimes for financial gain less necessary (Ramakers et al., 2017), but also has the potential to: provide a set of routines away from offending (Farrall, 2002; Laub & Sampson, 2003); increase self-esteem (Farrall, 2002); provide positive social bonds with co-workers and employers, and reduce time spent with criminally involved peers (Morrison & Bowman, 2017; Wright & Cullen, 2004); and provide an opportunity for generativity (i.e., to ‘give back’) (Maruna, 2001). Sampson and Laub (1993; see also Laub & Sampson, 2003) are particularly influential theorists in this realm and emphasise the importance of employment as a social bond to society and a source of informal social control. A stable job can generate social capital (i.e., social connections and associated resources and social benefits) and create a ‘stake in conformity’ so that individuals have more to risk and lose by reoffending. For desistance theorists more concerned with the role of agency and identity transformations in motivating and facilitating desistance, if viewed as a positive development, employment may also act as a ‘hook for change’ (Opsal, 2012). That is, it may act as a catalyst to foster or support cognitive transformations, particularly if it enables those desisting from crime to envisage or craft a satisfying ‘replacement self’ which is perceived to be fundamentally incompatible with further involvement in crime (Giordano et al., 2002).

However, as Owens (2009: 58) points out, there is something ‘overly general about the statement “employment promotes desistance”’. Research examining the link between employment/employment programmes and reduced recidivism has produced mixed results (Skardhamer & Savolainen, 2014: Ramakers et al., 2017) and it is unclear whether employment is a cause or consequence of desistance (Skardhamar & Savolainen, 2014). Different types of work have varied working conditions, pay, colleagues, skills, and promotional opportunities and, as such, are likely to present varying degrees of both appeal and opportunity to potential desisters (Owens, 2009). Indeed, desistance researchers have recognised the importance of meaningful or ‘quality’ employment to support desistance (with stability, adequate hours and pay, and a sense of work satisfaction) rather than the mere presence of a job (Leverentz, 2014; Österman, 2018; Sampson & Laub, 1990; Shover, 1996; Uggen, 1999). As Sampson and Laub (1990: 611) note, it is not merely employment, but ‘employment coupled with job stability, job commitment, and ties to work that should increase social control and, all else equal, lead to a reduction in criminal behaviour’. Similarly, Leverentz (2014: 141) comments, ‘[e]mployees need to be sufficiently well compensated to meet material needs and they need to be invested in the job for it to serve as a hook for change’. Uggen (1999) also found that, among a sample of released high-risk offenders, those who obtained high-quality jobs were less likely to reoffend than those who obtained lower-quality work. Shover (1996: 127) notes that ‘[n]ot all types of employment are equally likely to moderate offenders’ criminal involvement’. Rather, jobs most likely to facilitate desistance are those which ‘return a decent income, enable the individual to exercise intelligence and creativity, and allow for some autonomy in structuring the day’s activities’ (Shover, 1996: 127).

While quality, well-renumerated employment may support desistance, the types of work available to those with histories of imprisonment are often limited, and tend to be in low-wage, precarious positions with limited opportunities for personal or economic growth (Ricciardelli & Mooney, 2017; Sheppard & Ricciardelli, 2020). Such work may have a reduced desisting effect, and, under periods of financial strain, crime may be perceived as a more appealing way of making ends meet (Sheppard & Ricciardelli, 2020). Low quality, temporary jobs may not offer the same social control or access to bridging and linking social capital that can assist with overcoming structural constraints (Bracken et al., 2009) as more stable work, and workers in these roles may feel they have less to lose (or less of a ‘stake in conformity’) (Crutchfield, 1989, p. 495). In certain industries, individuals may be introduced to peer groups that encourage drug use, drinking and associated offending. In Godfrey et al.’s (2007) research, for example, for ‘occasional’ offenders, obtaining employment at a railway works meant immersion into a heavy drinking culture, which could lead to contact with the justice system.

### Barriers to Employment After Prison

Those leaving prison tend to face considerable obstacles to obtaining work generally, and especially rewarding, well-paid, stable work. They often have low educational attainment, high rates of prior unemployment, and limited work experience (Visher at al., 2011). Personal challenges in relation to mental health, trauma and addiction, initiated or exacerbated during their time in prison (Armour, 2012), may also hinder their ability to both obtain and maintain work (Anazado et al., 2017). Many leaving prison are also faced with restrictive release conditions, a lack of acceptable ID or a bank account, which can further complicate obtaining employment (Johnston, 2016). Additionally, they are likely to experience barriers to obtaining a suitable place to live (Mills et al., 2022), which is often necessary for desistance generally, as well as participating in work (Low et al., 2025).

A considerable barrier to employment is the stigma associated with a criminal record, especially for entry into high-status or career jobs (Western, 2007). Imprisonment creates gaps in employment history (a red flag to employers), and those leaving prison may lack familiarity with new workplace technologies, skills, processes and hiring practices (Anazodo et al., 2017). Thus, time in prison considerably limits individuals’ employment prospects; they are likely to struggle to find stable work, and employers may be unlikely to invest in them (Western, 2007). As such, they are often pushed into low-pay, part time, or temporary work with few (if any) opportunities for career development, and where they may be vulnerable to exploitation (Anazado et al., 2017; Western, 2007).

### Imprisonment and Employment in a Colonial Context

Opportunities for employment and desistance can differ substantially across socio-cultural contexts (Österman, 2018). Due to the ongoing, intergenerational legacies of colonisation, Māori, New Zealand’s Indigenous population, not only experience substantial criminalisation by the neo-colonial state, and consequent over-representation in the prison population,^i^ but also face significant barriers to obtaining well-paid, gratifying work. Māori were colonised by the British following the signing of Te Tiriti o Waitangi in 1840^ii^. Although the Treaty guaranteed Māori rights over their land and resources, the Crown breached its terms and introduced policies of systematic, large-scale theft of Māori land and forced cultural assimilation (Moewaka Barnes & McCreanor, 2019). Such policies not only led to Māori dislocation from ancestral lands and the undermining of Māori cultural practices and governance, but also the loss of Māori economic bases, creating long-term conditions of social, economic and political marginalisation (Jackson, 1988; Webb, 2017).

Increasingly Māori migrated into urban centres for work, where they typically faced limited employment opportunities, with long hours, low pay, challenging working conditions and little opportunity for economic growth, in addition to racism and discrimination in hiring and promotional practices (Poata-Smith, 2013). Neoliberal restructuring, the rolling back of the welfare state in the 1980s and 1990s, and the decline of the industrial and commercial sectors have furthered inequalities for Māori (Poata-Smith, 2013) and working class New Zealanders, leading to precarity; that is, ‘fundamental uncertainty of pay, conditions and duration of work’ which ‘includes work described as casual, zero hours, seasonal contracting (including labour hire) and fixed-term types of work’ (Wilson, 2014, p. 22).

In addition to economic marginalisation, Aotearoa New Zealand’s colonial history and neo-colonial structural violence have had other ongoing, intergenerational effects on Māori, including trauma; significant inequalities in relation to health, education, housing, and employment; and the normalisation of prison among many Māori communities (McIntosh & Radojkovic, 2012; George et al., 2014; Marriott & Sim, 2014). Māori continue to have an unemployment rate well above the national average (10.8% compared to 4.9%) (Ministry of Business, Innovation & Employment, 2021), with the average hourly wage for Māori employees being just 82% of the average hourly Pākehā^iii^ wage (The Treasury, 2018).

International literature has demonstrated that the relationship between desistance and employment is far from straightforward, but discussions of how employment can support or hinder desistance journeys in a colonial context remain limited, and it has been somewhat overlooked as a condition which may require ‘knifing off’ (Maruna & Roy, 2007) to sustain efforts towards desistance. The metaphor of ‘knifing off’ has been used in the desistance literature to describe the severance from situations, environments and social companions that may encourage or trigger criminal activity (Maruna & Roy, 2007). Separation from such environments and opportunities may enable the early stages of desistance if individuals are able to move away from the people, places or associations that increase the risk of engaging in crime, and more sustained desistance if they find new social environments and life situations, where they are not required to conform to a past identity and are able to develop clear future life scripts (Maruna & Roy, 2007). Drawing on narrative interviews with men leaving prison in Aotearoa New Zealand, this article therefore aims to explore some of the barriers to (or limitations of) the desisting effects of employment and whether certain jobs may need to be ‘knifed off’ for desistance efforts to be successful.

## Method

The interview data explored in this article derive from a wider project investigating the role of stable housing in reducing reoffending, funded by the Royal Society of New Zealand Marsden Fund. The larger project began with a quantitative interview study which followed a cohort of just over 200 people released from six prisons from the period just before release from prison to a year after release (see Mills et al., 2022). A small sub-sample from the quantitative study who had not reoffended within the last year and had indicated a desire to desist were invited to take part in a narrative interview to examine their post-release journeys and explore the role of stable housing and ‘home’ in facilitating desistance from crime. Participants had therefore entered at least the ‘primary’ phases of desistance (i.e., a period of non-offending) (Maruna et al., 2004). Although the wider research project had a particular focus on experiences of post-prison housing and ‘home’, many participants discussed employment during the interviews, signifying its importance to them which warrants further analysis and discussion.

Narrative interviews were held with 16 men between 14 and 20 months following their release from prison. At the time of the interviews, the men were aged between 26 and 68, with an average age of 44. Seven identified as Māori (with one of those men also identifying as Pākehā). The remaining nine men identified as Pākehā. Most described histories of sustained offending and serving multiple prison sentences. Offences they had committed included property offences, indecent exposure, drug dealing, domestic violence, wilful damage, wounding with intent to injure, drunk driving, aggravated assault and aggravated robbery. At the time of the interviews, 11 were working in some form of paid employment. Their fields of work included demolition and asbestos removal, farming, telemarketing, forestry, labouring, appliance repair, fruit picking, and work for a social services provider.

Narrative interviews provide a means of exploring people’s own subjective stories and experiences (Anderson & Kirkpatrick, 2015). Participants were asked about their experiences before and after imprisonment, and key factors which had helped them to not return to prison. Questions included, ‘Could you tell me about your life before going to prison?’ and ‘What do you think has helped you not to return to prison?’ The interviews took a broad approach and invited participants to raise factors that had been significant to them in their desistance journeys. In contrast to more structured interview approaches, narrative interviews prioritise the story teller’s perspective and ‘let the interviewee control the direction, content and pace of the interview’ (Anderson & Kirkpatrick, 2015: 1).

Interviews took place in a location convenient for participants, including their current accommodation or a local café, and lasted between one and two and a half hours. At the start of the interview, the interviewer went through the participant information sheet and gave participants the opportunity to ask any questions to ensure that each participant made a free and informed decision to take part. After the interviewer had answered any questions, participants signed a consent form. Participants were offered a koha^iv^ to thank them for their time and contribution to the research. Ethical approval for the research was granted by the University of Auckland Human Participants Ethics Committee.

With participants’ consent, each interview was recorded and transcribed verbatim. The data were analysed according to a thematic approach using NVivo qualitative data analysis software. Thematic analysis is a method of identifying and organising patterns of meaning (themes) across the data (Braun & Clarke, 2012). The first and second authors read and re-read the transcripts to identify key codes concerning what had contributed to the men’s pathways in and out of the justice system, and any barriers that prevented them from making a change. In some cases, differences in coding practices led to further discussions between the two coders before a consensus was reached. The initial codes were then compared both within and across interviews to establish the key themes, such as employment, and sub-themes, such as barriers to employment, employment disincentives, the colonial context, employment as a pathway into offending and the importance of the right type of work for desistance. These sub-themes form a structure for the discussion of the findings below.

## Findings

### Criminal Records as Barriers to Meaningful Work and Career Progression

For eleven of the 16 men in the sample, a considerable barrier to obtaining work – and particularly the well-paid, gratifying work that they desired – was the stigma associated with their criminal records. A study of New Zealand industries found that just over a third (34.4%) would not hire someone with an offending history (Gilbert et al., 2019). The men were acutely aware of this barrier. As Tama noted:

There’s not enough employers out there that are forgiving of somebody that’s been inside. It’s like they’re pre-judged, and pretty much everybody gets that these days, and you can’t hide anything. Some employers out there, and even with just small things, they’re just not interested. (Tama)

A criminal record could also pose a barrier to career progression and the potentially desisting effects of rewarding, higher-pay, work. Charlie, for example, had returned to work in asbestos removal, but wanted to advance into a management position. He explained how his criminal record held him back from this promotion:

I’ve got a potential career to be an operations manager, I can go and sign my contract now, but when I comes to the MOJ [criminal record check], I know I’m fucked [. . .] I want to get managerial. And how do you get there when you’ve got 15 years prison under your belt? I’m qualified to do all that but all I need is that one shot to prove myself. (Charlie)

Even semi-legitimate jobs which paid cash in hand could be scuppered by the presence of certain convictions, such as arson, particularly if a former employer was the target of the offending:

No-one in their right mind is actually going to give me a job because the past catches up. I’ll give you an instance. This last week a dude turned up who sells second-hand ovens and stuff like that [. . .] So I was telling him that I was semi-retired [. . .] About 10 minutes later he said, “I’ve got oven cleaning,” under the table and that [. . .] I rang him a couple of days later, but I could hear the difference. He would have been to see [ex-employer] and [ex-employer] would have told him that I torched [name of firm] [. . .] Really shot myself in the foot with that. (Sam)

However, the barrier of a criminal record was not always insurmountable. As Nick suggested, it could be more beneficial to not immediately signal a criminal record to employers, but instead talk to them about the nature of the conviction during the recruitment process:

A lot of people are just downtrodden because of the consensus of ‘you can’t get a job with a conviction’, but you can. If you go for an interview and you get the job, and they turn around and say, “No”, because they’ve found something on you, or they’ve decided to ask that question, then have that conversation. Have it when they ask, and don’t go in there and be like, “Hi, I’m a burglar and I eat children.’ (Nick)

Others were upfront about their record and were still able to find work, though this was often confined to seasonal, manual labour:

Interviewer: Has you having been in prison ever stopped you from getting a job?

Pene: No. ‘Cause I just tell them straight up. I just tell them, “I’ve been to prison, but I bet you I can work harder than half of these other dudes”.

### State Welfare and Low Pay as Employment Disincentives

Another substantial barrier to employment is the operation of the state welfare system in Aotearoa which meant that for some participants, it was simply not worth working. As Jacob recounts, by substantially reducing the amount of benefit received, the system could obstruct participation in paid employment:

I was doing work in the kiwifruit [picking industry]. [. . .] I declared my hours with Work and Income and that was such the wrong thing to do. . . They chopped my benefit by like $200. They paid $150 to my rent and that was it. . . So I had to cover whatever else, my bills and everything from my 13 hours’ worth of wages. . . it actually put me $137 in debt with my landlord, [and] left me with less than what Work and Income gives me in a week. I asked them for assistance, and they said, “No. Go and work for more.” Because they made me short that week, I couldn’t go back to work to earn money. They weren’t gonna give me gas money. (Jacob)

When those leaving prison are only able to access low paid work, they may not gain enough of a financial reward to leave the benefit, creating a financial disincentive to employment (Singley, 2003). This was also evident in Rob’s case:

I told the boss that I was working for, I said ‘Bro, it’s not working out. I can’t work here anymore. I’m not quitting, you’re not paying me enough, I get more on the benefit’ (Rob)

Consistent with previous international research, those leaving prison in Aotearoa New Zealand face numerous barriers to securing well-paid employment and career advancement, thereby limiting the potential impact of employment on desistance.

### Colonial Context and Racism

Many stories from Māori participants were reflective of the ongoing, intergenerational effects of colonisation, including considerable socioeconomic disadvantage and the normalisation of prison (McIntosh & Radojkovic, 2012). In such contexts, the men spoke of few (if any) avenues into conventional education or stable, well-paid employment, and many described ‘rough’ childhoods, characterised by exposure to violence, drugs, and gangs. Rob, for example, described his offending and drug use as being shaped by his upbringing and the hardship he faced even before birth: ‘I was already in this rough environment before I was even born, so I was already influenced and all of that while I was still in my mum’. These men often described prison as a normal or inevitable part of life. As Nikau said: ‘I started going to the little jails in the cop-shop, that started pretty young. That was just inevitable, eh.’ Rawiri referred to his involvement in crime and prison as ‘fate’ and a ‘legacy’.

Many of the Māori men grew up in environments of considerable hardship and lacked the bridging and linking social capital and support which may have paved a route to education or meaningful employment as an alternative to prison. Consequently, after prison, the majority were further excluded from stable, well paid, meaningful employment and, in some cases, from the job market altogether. This exclusion appeared to be compounded by prejudice. Rawiri, for example, commented on the racism and discrimination he had experienced at work: ‘Some of the jobs, there was racism. And I just didn’t tolerate it.’ He added that for Māori: ‘life itself will always be hard, like the system. It’s always going to be pretty rough’.

At the time of the interview, Rawiri was not working in paid employment and was receiving financial assistance from the state (‘the benefit’). Like prison, he referred to the intergenerational nature of state financial assistance: ‘I’m on the benefit, that’s a legacy itself’. However, Rawiri and other Māori men in the sample spoke of having little trust or faith in the government or ‘system’. Nikau spoke of Māori becoming ‘lost in the system’ and the detrimental impacts this could have on Māori sense of cultural identity. He connected this loss of identity to Māori imprisonment and state financial assistance (the ‘dole’):

We seem to have lost ourselves, especially with the system and how it’s running. It’s like we’re getting taken away from our identity [. . .] I think a lot of us get lost eh; especially Māori I see in prison. They’ve lost themselves. It’s hard for them to find themselves. Too used to getting the dole and buying drugs. (Nikau)

Māori leaving prison are re-entering a colonial society (Mills & Lindsay Latimer, 2021) where they not only face the stigma of a criminal record but are vulnerable to racism and further discrimination from employers (Tan et al., 2024), making it substantially more challenging for them to obtain the kind of employment that is likely to support the desistance process.

### Employment as a Pathway into Crime Rather Than Out

While employment is often touted for its desisting effects, as a source of (pro)social capital (Farrall, 2002; Sampson & Laub, 1993; Wright & Cullen, 2004), for some of the men in the sample, employment embedded them deeper into antisocial networks and offending/drug-using lifestyles. Pene described ‘getting into trouble’, ‘drinking and fighting’ while working as a sheep shearer. He explained: ‘it was in the shearing environment. They’re pretty into their beer, and then I just started picking it up.’ Similarly, Toby discussed how the work culture in forestry led him to smoking methamphetamine:

A lot of it was work culture – drinking and drugs and shit in the work culture. We had a guy in our crew who was dealing the shit quite heavily. He would feed us up at work [. . .]. Then after work we’d get paid and we’d be like, “Need some more.” So, it would be straight off to his place and go and get some more. He was gaining off us, by shouting us a little bit [. . .] Then it just progressed from there. (Toby)

When Charlie left prison on a previous occasion and obtained employment working in asbestos removal, he was introduced to a new social network of drug-using co-workers. While co-workers are often spoken of as a source of support and social capital (e.g., Wright & Cullen, 2004), in Charlie’s case, ‘knifing off’ his job and co-workers appeared necessary for his desistance, even though this meant losing his only source of income and a well-paid job:

I just come to a point where, fuck, I’d had enough. I quit my job [. . .] All the employees were into it [drugs]. I got to cut loose, I got to get straight. [. . .] $1200 bucks a week easy money, but I cut it to get away from all the drugs and everything. (Charlie)

It was not only Charlie’s co-workers that encouraged his drug use, but also the work itself. He explained that the demanding nature of the work, and the associated lack of sleep, led to drug use in order to ‘keep going’:

I was doing 92-hour weeks. You needed that shit [drugs] just to keep going [. . .] Shit four nights sleep in six weeks, that’s fucken not bad. I lost a load of weight, I was smashing jobs out [. . .] Get your bonuses, money, money, drugs, money, work. (Charlie).

Charlie and other participants also discussed dangerous working conditions which could put their physical health at risk. These jobs did not appear to provide a sense of purpose or fulfilment in support of the men’s desistance, but could be well-paid and necessary to make ends meet:

I do it [demolition work] if I’m really, really stuck for a dollar. I just hate doing it, I really do. I can do it [. . .] But to be sucking in contaminants all day and injuring yourself . . . strip out demolition is really, really physical and it’s a young man’s job and I prefer not to be doing it. (Sam)

I’ve spent 10 years in this bullshit industry [asbestos removal], that’ll kill me, well asbestos will kill you and there is no way you can do this job 100% without getting fucked up. (Charlie)

Other men in the sample spoke of the detrimental effects that particular types of work, including white-collar work, could have on their mental health and wellbeing. Nick described working night shifts at an energy company, and the negative impact this had on his sleeping patterns. This, in turn, tempted him to revert to drugs and he left his job to avoid this:

I did actually get a job with [energy company] [. . .] but I didn’t want it, I had to work graveyard shifts and stuff. I was pretty much employed to work from midnight until seven.[. . .] I will struggle to sleep as it is, but shifting my shifts around like that I felt I was going to end up close to drug use [. . .] so I went unemployed again. (Nick)

Like Charlie’s, Nick’s desistance and wellbeing appeared to be supported by ‘knifing off’ a previous job, and instead seeking employment with more manageable hours. Dave also commented on the detrimental effects the wrong type of work could have on his health. Unemployed at the time of the interview, he explained how his prior experiences of employment had led to mental breakdowns, depression, drug use and eventually imprisonment:

I’ve ended up mentally unstable from it [work]. I’ve had proper breakdowns and ended up in mental institutions and ended up in jail and stuff like that. [. . .] If I force myself to do it [work] I can, but it just makes me worse. I start getting depressed about it ‘Oh, I have to do this’ so I go and do it and then I’m like upset. (Dave)

Particular types of work could lead to social networks that encouraged drug use, or challenging working conditions which could lead to mental and physical health issues and associated drug use. Drug use can in turn impair judgement, decision-making and impulse control, leading to greater risks of involvement in reckless and violent behaviour, and those using drugs may also engage in property crime to finance their drug habits (Gilbert, 2024). For some of the men, it was therefore the ‘knifing off’ (Maruna & Roy, 2007) of the job itself which supported their desistance, rather than the job acting as a ‘hook for change’ and an opportunity to craft an alternative, satisfying future self (Giordano et al., 2002).

### The right ‘type’ of work as motivation for desistance

Despite the considerable barriers that the men often faced to obtaining stable, well-paid, meaningful employment and higher positions, they still recognised the value of employment (or a particular type of employment) for supporting the desistance process. Some had managed to find work that provided them with a sense of meaning and purpose, while others were not currently employed or working in what they considered to be a fulfilling job, but they could envisage the right ‘type’ of employment (Oswald, 2023) as an opportunity to promote their desistance and a more positive future.

Consistent with existing desistance literature (e.g., Farrall, 2002), some of the men recognised the benefits of employment for their routine activities, dominating their daily schedules and leaving little time or energy to get into trouble: ‘You just can’t get in trouble when you’re working [. . .] That’s one thing about keeping busy, keeps you out of trouble’ (Kevin). Others spoke of supportive co-workers and bosses who could act as a source of both social capital and informal social control to keep their desistance in check (Laub & Sampson, 2003). In some cases, they had worked for their employer prior to prison for several years and were able to maintain these well-established social connections. John, who had recently received a promotion at the time of the interview, had worked on and off for the same boss at a demolition company since he was 19. He spoke of the social control his boss exerts over his behaviour:

He came to visit me when I was in jail and he’s just always been really supportive, but also told me to fuck off when I’ve been useless or using. He’s fired me about four or five times. He’s a good support but won’t put up with bad behaviour as well. (John)

Although now semi-retired, Sam had known his former employer, Harry,^v^ for 16 years. In that time, he had not only re-employed Sam on several occasions, but had also become a highly valued friend, who actively supports Sam’s desistance:

I got out of Dunedin Prison and my mate, Harry, had got a hold of me to say that he was starting a demo firm, he wanted me to come and work for him. We met years ago in demolition. He was always a good friend. . . he gets me a lot of work and he’s always tried to save me from me [. . .] I love him to pieces. He’s a great friend. He’s not malicious. He’s not judgemental [. . .] He’s just such a lovely man. (Sam)

However, the men emphasised the importance of having the right ‘type’ of work to support their desistance, rather than any job. Nick was working in a tele sales role for a mobile phone company. While previous jobs had had a detrimental impact on his mental health (as discussed above), his current job appeared to promote a sense of wellbeing in support of his desistance. His association with the company brand provided him with a sense of pride: ‘I’m actually quite happy when I write an email to see my name on there, signature and stuff. It’s a bit nerdy, but yeah, I do like the brand’. Nick’s job also provided him with social connection and purpose; he no longer relied on drugs as a ‘substitute’:

I’m really happy with my job at the moment [. . .] I’m not feeling the enthusiasm for using, because I’m socially connected and doing something with myself. And so, I’m not having to substitute that with something synthetic that strings me out for days. (Nick)

Several of the participants spoke about the need to provide people in prison with better training and education opportunities, including the chance to learn a trade to enable them to access purposeful, meaningful work. Toby, for example, commented on the poor-quality work available through schemes such as Release to Work,^vi^ and emphasised that trade training and meaningful work with good remuneration would provide a social bond to society (Laub & Sampson, 1993), and prevent people from returning to prison:

Why not get these people into proper trades? Teach them how to weld. Teach them how to build. Actually, while they’re inside give people trade opportunities, [. . .] so when they get out, they’ve got a qualification, so they don’t need to go back to the life they live, because they’ve got something they enjoy doing, which is going to pay them well. (Toby)

Charlie similarly emphasised the importance of providing rewarding work opportunities and training for young men leaving prison and suggested that if he had access to such opportunities, his own pathway would have been different: ‘‘Cos if that was in place for me, I would have strived and actually made something instead of just earning [gang] patches.’ He reflected on his time in prison, wishing he had had educational opportunities to turn his life around:

15 years inside mate, that’s a long time. That could be training, that could be education, [. . .] the only education I got in there was knuckle up, you know? [. . .]. If I could turn back time I’d get an education and stay the fuck away from gangs [. . .] Instead of getting educated in dealing drugs, stealing cars, guns, firearms. (Charlie).

Thus, it appeared the men needed a particular ‘type’ of work to support their desistance and wellbeing, rather than any job (Oswald, 2023). Ungratifying, temporary positions, with limited opportunities for growth and supportive social connections, and potentially dangerous working conditions, are unlikely to exhibit the same (if any) desisting effects as more gratifying work (Sheppard & Ricciardelli, 2020).

## Discussion

Employment has been commended for its ability to trigger or support desistance (Maruna, 2001; Sampson & Laub, 1993), but the current study has explored some of the negative impacts of employment, and the mechanisms by which employment, even that which is well-renumerated, can encourage recidivism rather than support desistance. While existing research has emphasised that employment can provide an opportunity to associate with law abiding peers and to move away from ‘deviant’ peers and associated pressures (Wright & Cullen, 2004), consistent with differential association theory, the current research found that employment could hinder desistance by providing a gateway to ‘deviant’ peers and lifestyles. For some, employment was therefore a source of anti-social capital rather than pro-social capital, which lacked the potential to form constructive social bonds and/or act as a form of informal social control (Sampson & Laub, 1993). Furthermore, the work itself could lead to drug use to cope with the demands of the job, and certain types of work, such as shift work, could have detrimental impacts on the men’s physical and mental health and wellbeing, leading them to revert to substance use and/or offending, hindering or potentially hindering their desistance journey. Employment has received little attention in discussions of the concept of ‘knifing off’ and its contribution to desistance (Maruna & Roy, 2007), but for some participants, such as Nick and Dave, it was necessary for them to actively distance themselves from types of work or work environments to avoid becoming their ‘feared selves’, which can be a powerful motivator in the early stages of desistance (Paternoster & Bushway 2009).

The setting of the study in the colonial context of Aotearoa New Zealand, has enabled an investigation into some of the barriers experienced by Māori men, to employment and potential desistance. Whilst many participants had experienced challenges obtaining employment as a result of their criminal record, Māori participants faced additional structural constraints to obtaining employment due to the ongoing effects of colonisation, including socioeconomic disadvantage, a lack of bridging and linking social capital to support them into work, the normalisation of prison, direct racism from employers, and an understandable lack of trust in the ‘system’ (i.e., government agencies). Some of the men reflected on how their experiences of social exclusion had led to them (and similarly situated Māori men) becoming ‘lost in the system’, losing their sense of self and connection to their cultural identity. The limited desistance literature involving Indigenous populations emphasises the importance of cultural identity in fostering desistance (Bracken et al., 2009; Richards et al., 2020). The experiences of the men in this research suggest that neo-colonial policies and practices of social exclusion and economic marginalisation have actively worked against the construction of more positive cultural identities to support their desistance, limited their access to conventional pathways into education or stable, rewarding employment, and worked against their efforts towards desistance, although all were desisting at the time of the interview. Glynn (2014) has similarly found Black men leaving prison in the UK and the US were re-entering a society where the social structure and criminal justice systems are highly racialised, significantly limiting meaningful and productive opportunities for Black men to exercise agency and desist from criminal behaviour. The current findings are based on a small sample of Māori men in Aotearoa New Zealand, but further research could explore whether their experiences are reflective of Indigenous populations in other settler states, who have similarly been marginalised through colonial policies and practices (Cunneen, 2009). Such research could adopt an intersectional approach (Glynn, 2016), examining how multiple interlocking oppressions may constrain desistance.

This research also emphasises the importance of the right ‘type’ of work to support desistance, rather than work generally (Oswald, 2023). Some participants had found employment which they perceived as motivating and meaningful, and this work could provide them with social connections, purpose, and an opportunity to craft an alternative sense of self. However, many available jobs were of a precarious nature, involving long-hours and, on some occasions, dangerous working conditions which were unlikely to support their desistance. Many participants recognised that alternative types of work such as skilled trades with good remuneration, a sense of work satisfaction and progression could act as a ‘hook or change’ to craft a ‘future self’ (Giordano et al., 2002; Paternoster & Bushway, 2009).

The findings therefore suggest that to support desistance, those who are able and willing to engage in employment should not be encouraged into ‘any job’ upon release from prison. Rather they should be supported into desirable work which can provide adequate remuneration and a sense of satisfaction. As participants suggested, opportunities in prison to learn a trade could help them to obtain the skills necessary to obtain more rewarding employment. International research regarding the efficacy of prison vocational education in reducing recidivism and improving post-prison employment outcomes is somewhat mixed and existing studies frequently do not account for selection bias which may inflate the apparent success of such programmes (McNeely, 2023). Nevertheless, such programmes can represent important pathways for individuals who are highly motivated to change to meet their career goals (McNeely, 2023). While the Department of Corrections (2024) states that they offer ‘a range of qualifications, training and support’, including ‘industry qualification training’, the men’s experiences suggest that these services are not widely available or accessible. Programmes such as Release to Work, where minimum security prisoners engage in paid work in the community to help them obtain employment on their release (Department of Corrections, 2021) can help to overcome some of the barriers to employment in the post-release period (Johnston, 2016), however, these initiatives often involve low-paid, repetitive work and are only available to a select group of prisoners. As found in this study, pre-prison employers serve as a valuable source of social capital and social control. Existing research has shown that those who return to a pre-prison employer are more likely to secure full-time, permanent work and to be ‘doing well’ in other aspects of reintegration (Morrison & Bowman, 2017) and are less likely to reoffend (Ramakers et al., 2017). It may therefore be beneficial to reach out to current employers when someone is incarcerated to ascertain if they would be willing to reinstate the person’s employment upon release.

All prisoners who need and desire employment assistance would benefit from access to their own individualised tailored training and employment support plan to develop the skills they require to obtain a desirable, meaningful job on release. Currently Employment and Training Consultants can assist prisoners to obtain relevant training and education but only work with prisoners who are within four months of release, potentially leaving little time to engage in training that will lead to rewarding, high-quality employment. An evaluation of the Minnesota Department of Corrections EMPLOY programme, which provides employment assistance from the last few months of imprisonment to one year after release, has suggested that prisoner employment programmes are more likely to reduce recidivism and lead to successful employment outcomes if they provide a continuum of support during imprisonment and after release in the community (Duwe, 2015). It is currently unclear whether Employment and Training Consultants provide assistance beyond the period of imprisonment.

The Department of Corrections could partner with career training institutions and employers to improve access to high quality training and stable employment opportunities. A good example of such an initiative is Take2, a not-for-profit organisation, which teaches prisoners technology and life skills, and works with employers to assist them to obtain high-quality, well-paid jobs in the technology sector after their release. The Take2 programme includes workshops from leading industry professionals and well-known motivational speakers, and a 24-month reintegration programme during which each participant is given a mentor from the industry (Wiggill, 2024). A recent study involving Take2 graduates found that by acting as a ‘hook for change’, the Take2 programme supported a change in self-identity and therefore desistance from crime (Wiggill, 2024). However, this programme currently only runs in one prison in Aotearoa New Zealand, so access is limited to a small group of prisoners.

Even if the stigma of a criminal record does not prevent participants from obtaining work, it can hinder opportunities for career progression. New Zealand’s Criminal Records (Clean Slate) Act 2004 enables convictions to be concealed if an individual has never received a custodial sentence and has no fresh convictions within the last seven years. Those who have been incarcerated are prevented from ever concealing their record, irrespective of their offence, sentence, or the period of time since they were in prison (Johnston, 2016). They may be forever labelled as ‘ex-offenders’, posing challenges to their likelihood of ‘secondary desistance’ (i.e., an identity change associated with non-offending) as well as tertiary desistance (a feeling of community acceptance and belonging) (Maruna et al., 2004; McNeill, 2016).

International research suggests that the recidivism risk of a person convicted six or seven years ago begins to approximate the risk of people with no criminal record (Kurlychek & Bushway, 2006). In line with international clean slate schemes (such as the one adopted in England and Wales), the Clean Slate scheme could therefore be reformed to include those who have served time in prison (Low, 2024) and enable them to apply for a concealment of their criminal record. These changes would help to remove the stigma of a criminal record as a barrier to employment generally, and to more meaningful, gratifying work with opportunities for career advancement. Additionally, the state welfare system could be reformed to focus on enabling and incentivising those who seek and obtain employment, rather than discouraging them from doing so through substantial reductions in benefit entitlements.

Finally, the findings in this research emphasise the state’s responsibility to provide redress for its contemporary and historical wrongdoings against Māori, including the necessary resources and support to nurture, rather than stifle, Māori desistance journeys. This would involve providing support to break down the socio-structural barriers that Māori often face to desistance generally, and to obtaining high quality work, including the provision of suitable housing, assistance to re-connect with whānau^vii^, mental health support, addiction treatment, and educational/training opportunities. Given the men’s limited trust or faith in the government, these services should be Māori-led (run by Māori, for Māori) and could also include initiatives which focus on healing from the ongoing effects of intergenerational trauma, including the effects that this can have on a sense of self and identity (Bracken et al., 2009; Wirihana & Smith, 2014).

## Conclusion

This research has highlighted various barriers to the desisting effects of employment among a sample of men leaving prison in Aotearoa New Zealand and has emphasised the ways in which the ongoing effects of New Zealand’s colonial history has posed barriers to Māori obtaining rewarding and meaningful employment. The study has emphasised the ways in which differing types of work can have varying effects on desistance, with some types of work significantly hindering the desistance process, negatively affecting wellbeing and encouraging recidivism, ensuring that, for some, the only way to continue to their desistance journey was to ‘knife off’ their employment entirely. Thus, in order to support the desistance process, it is important that policy and practice recognise that those leaving prison should not be encouraged into any job, rather they require support from employers, the state and other agencies to enable them to access work that provides them with adequate remuneration, satisfaction, and can help them to build a positive sense of self to foster their desistance.

## Notes

i Despite representing only around 17% of the general population, Māori comprise 53% of the prison population (Department of Corrections, 2022; Statistics New Zealand, 2020)

ii The founding document of Aotearoa New Zealand

iii Māori language term for New Zealanders of European descent

iv Gift

v Names have been changed to ensure anoymity

vi A scheme to enable minimum-security prisoners to access work in the community during their prison sentence (Department of Corrections, 2021).

vii Extended family and networks of support.
